# Response to: Comment on “Gut Microbiota as a Driver of Inflammation in Nonalcoholic Fatty Liver Disease”

**DOI:** 10.1155/2018/7328057

**Published:** 2018-08-19

**Authors:** Stefano Bibbò, Maria Pina Dore, Giovanni Cammarota

**Affiliations:** ^1^Department of Clinical, Surgical and Experimental Sciences, University of Sassari, Sassari, Italy; ^2^Department of Gastroenterology, Catholic University, School of Medicine and Surgery, A. Gemelli Hospital, Rome, Italy

We would like to thank Li and Yuan for their insightful comments [1] on our article [2]. The authors focused on the role played by gut microbiota in the pathways of nonalcoholic fatty liver disease (NAFLD) and proposed that the modulation of intestinal dysbiosis may alter the progression of the disease. We agree with this suggestion; indeed, several studies reported the beneficial effect of gut microbiota modulation on NAFLD patients. Studies published in the last year demonstrated that the administration of multistrain probiotic mixtures improved clinical outcomes in overweight patients with NAFLD, normalization of markers of a chronic systemic inflammatory state [3], or the shift towards a normal pattern of fecal gut microbiota [4]. Furthermore, therapeutic modulation of gut microbiota has also proved to be effective in lean patients with NAFLD [5], maybe suggesting a role for gut microbiota independent of adiposity.

Finally, fecal microbiota transplantation (FMT) was also considered a therapeutic tool in NAFLD. Very intriguing results were reported in a trial in mice, showing the reduction of intrahepatic fatty accumulation and of proinflammatory cytokines [6]. Unfortunately, to date, evidence on the beneficial effects of FMT on humans is lacking.

In conclusion, we hope that gut microbiota modulation will be considered a therapeutic tool in patients with NAFLD, but more studies are needed.
